# TCA Cycle and Fatty Acids Oxidation Reflect Early Cardiorenal Damage in Normoalbuminuric Subjects with Controlled Hypertension

**DOI:** 10.3390/antiox10071100

**Published:** 2021-07-09

**Authors:** Aranzazu Santiago-Hernandez, Marta Martin-Lorenzo, Ariadna Martin-Blazquez, Gema Ruiz-Hurtado, Maria G Barderas, Julian Segura, Luis M Ruilope, Gloria Alvarez-Llamas

**Affiliations:** 1 Immunology Department, IIS-Fundación Jiménez Díaz-UAM, 28040 Madrid, Spain; aranzazu.santiago@quironsalud.es (A.S.-H.); marta.martin@fjd.es (M.M.-L.); ariadna.martinb@quironsalud.es (A.M.-B.); 2 Cardiorenal Translational Laboratory, Institute of Research i+12, Hospital Universitario 12 de Octubre, 28041 Madrid, Spain; gemainfantes@yahoo.es (G.R.-H.); ruilope@icloud.com (L.M.R.); 3 CIBER-CV, Hospital Universitario 12 de Octubre, 28041 Madrid, Spain; 4 Department of Vascular Physiopathology, Hospital Nacional de Parapléjicos, SESCAM, 45071 Toledo, Spain; megonzalezb@sescam.jccm.es; 5 Hypertension Unit, Hospital Universitario 12 de Octubre, 28041 Madrid, Spain; hta@juliansegura.com; 6 European University of Madrid, 28108 Madrid, Spain; 7 REDINREN, IIS-Fundación Jiménez Díaz, 28040 Madrid, Spain

**Keywords:** albuminuria, chronic kidney disease, normoalbuminuria, high-normal, cardiovascular risk, Hypertension, tricarboxylic acid cycle, free fatty acids, β-oxidation, metabolomics

## Abstract

Moderately increased albuminuria, defined by an albumin to creatinine ratio (ACR) > 30 mg/g, is an indicator of subclinical organ damage associated with a higher risk of cardiovascular and renal disease. Normoalbuminuric subjects are considered at no cardiorenal risk in clinical practice, and molecular changes underlying early development are unclear. To decipher subjacent mechanisms, we stratified the normoalbuminuria condition. A total of 37 hypertensive patients under chronic renin–angiotensin system (RAS) suppression with ACR values in the normoalbuminuria range were included and classified as control (C) (ACR < 10 mg/g) and high-normal (HN) (ACR = 10–30 mg/g). Target metabolomic analysis was carried out by liquid chromatography and mass spectrometry to investigate the role of the cardiorenal risk urinary metabolites previously identified. Besides this, urinary free fatty acids (FFAs), fatty acid binding protein 1 (FABP1) and nephrin were analyzed by colorimetric and ELISA assays. A Mann–Whitney test was applied, ROC curves were calculated and Spearman correlation analysis was carried out. Nine metabolites showed significantly altered abundance in HN versus C, and urinary FFAs and FABP1 increased in HN group, pointing to dysregulation in the tricarboxylic acid cycle (TCA) cycle and fatty acids β-oxidation. We showed here how cardiorenal metabolites associate with albuminuria, already in the normoalbuminuric range, evidencing early renal damage at a tubular level and suggesting increased β-oxidation to potentially counteract fatty acids overload in the HN range.

## 1. Introduction

Microalbuminuria, or moderately increased albuminuria according to recent guidelines, is defined as an albumin to creatinine ratio (ACR) over 30 mg/g in urine. It is a marker of glomerular and tubular injury and an indicator of subclinical organ damage. As such, moderately increased albuminuria is an established risk factor of cardiovascular morbi-mortality and renal disease, particularly, but not exclusively, in hypertensive or diabetic subjects [1,2], and its added value has been proposed in cardiovascular risk stratification [3,4].

Recent clinical evidence points to an association between cardiovascular risk and renal function decline with urinary albumin concentration already in the normoalbuminuria range (ACR < 30 mg/g). Cardiovascular events [5,6], increased incidence of chronic kidney disease (CKD) [7] and a faster decline of estimated glomerular filtration rate (eGFR) has been reported for normoalbuminuric subjects within the high–normal range (ACR = 10–30 mg/g) [8]. In normoalbuminuric diabetic kidney disease patients, quantitative detection of albuminuria has been proposed together with clinical follow-up for cardiovascular events for those subjects with a urinary albumin excretion rate of 10–29 mg/24 h [9].

Despite these potential fatal clinical outcomes, normoalbuminuric subjects are usually considered at no cardiorenal risk in daily clinical practice, and molecular changes underlying early development are still unclear. In a relevant subgroup of hypertensive patients under chronic suppression of the renin–angiotensin system (RAS), progression from normoalbuminuria to moderately increased albuminuria was observed despite optimal blood pressure control [10]. In this line, previous research from our group revealed a urinary metabolic signature associated with albuminuria development in those hypertensive subjects [11]. Particularly, we have shown in further studies that four metabolites (oxaloacetic acid, 3-ureidopropionic acid, guanidoacetic acid and malic acid) already had an alteration in normoalbuminuric patients within the high–normal range compared to those with ACR < 10 mg/g [12]. In other metabolic studies, uremic toxins and molecules related to carnitine metabolism were suggested as predictors of incident microalbuminuria in type 1 diabetic subjects [13]. The trimethylamine N-oxide (TMAO) pathway is related to intestinal microbiota and has been associated with cardiovascular risk. In particular, TMAO modulates lipid metabolism and increases vascular cell activation and endogenous inflammation, promoting atherogenesis. In type 2 diabetic subjects with albuminuria, the combination of a sub-set of TMAO pathway metabolites were shown to be associated with a higher risk of renal function decline [14]. Tricarboxylic acid cycle (TCA) metabolites also showed a prediction capacity for CKD progression in individuals with type 2 diabetes, independent of traditional cardio–renal risk factors, including eGFR and albuminuria [15]. These studies prove the capacity of metabolic profiling to reflect pathophysiological alterations subjacent to albuminuria development, even in the early stage.

In order to better decipher the underlying mechanisms involved and to help in elucidating novel routes of action for early prevention, here we stratified the normoalbuminuria condition in terms of ACR, with the aim of investigating the potential role of urinary metabolites previously identified in association with cardiorenal risk.

## 2. Methods

### 2.1. Patients Selection and Urine Samples Collection

A total of 37 hypertensive patients under chronic RAS suppression, with ACR values in the normoalbuminuria range (<30 mg/g), were recruited at the Hypertension Unit in Hospital 12 de Octubre (Madrid). Patients with or without diabetes mellitus between 40 and 75 years were included. Subjects with an estimated glomerular filtrate (eGFR) below 60 mL/min/1.73 m^2^ or with secondary hypertension were excluded. Blood pressure values, estimated as the mean of three readings, were measured at the office with a validated semiautomatic oscillometric device, after 5 min resting in a sitting position. Subjects were classified according to their basal ACR urine values into a control group (C), including subjects with ACR < 10 mg/g, and high–normal group (HN), including subjects with ACR = 10–30 mg/g.

Urine samples were collected in sterile recipients, centrifuged and stored at −80 °C until analysis, as previously published [12,16,17].

### 2.2. Metabolic Analysis by Targeted Mass Spectrometry

Based on previous studies from our group, we selected a panel of metabolites associated with cardiorenal risk in terms of atherosclerosis development, acute coronary syndrome at the onset and existence of cardiovascular risk factors, including moderately increased albuminuria, CKD or resistant hypertension [11,16,17,18,19,20]. Supplementary Table S1 summarizes the selected metabolites from previous findings to be analyzed in patients within the HN range, in comparison to those with ACR < 10 mg/g. Metabolite analysis was carried out by liquid chromatography coupled to mass spectrometry (LC-MS/MS) in the selected reaction monitoring (SRM) mode, as previously published. Mass Hunter Software (v4.0 Agilent Technologies, Santa Clara, CA, USA) was used to control a 6460 Triple Quadrupole and 1200 Series HPLC system (Agilent Technologies). Briefly, the urinary protein fraction was eliminated by organic precipitation, and metabolites’ chromatographic separation took place at 0.4 mL/min in an acetonitrile gradient in positive or negative ion mode. Optimal conditions of analysis were first established with commercial standards and are detailed in Supplementary Table S2. Individual signals were integrated by Mass Hunter Qualitative Analysis software (v4.0 Agilent Technologies), and peak areas were calculated. Metabolite abundances were normalized by the urinary creatinine value.

### 2.3. Analysis of β-oxidation Targets: Urinary Free Fatty Acids, Liver Fatty Acid Binding Protein and Nephrin

Urinary free fatty acids (FFAs) concentration was calculated using a colorimetric Free Fatty Acid Assay (Abcam, ab65341, Cambridge, UK). Concentration values were expressed in nmol/µL. Urine human liver fatty acid binding protein (FABP1) was analyzed by an ELISA assay (High Sensitive ELISA Kit for Fatty Acid Binding Protein 1, Cloud-clone, HEB566Hu-96T). Estimated values were expressed in ng/mL. Nephrin protein was measured in urine by an indirect ELISA assay (Human Nephrin ELISA, Ethosbiosciences, 1037), and concentration values were expressed in µg/mL.

### 2.4. Statistical Analysis

Significant differences in clinical characteristics, medication, metabolites abundance, FFAs and protein levels between C and HN groups were evaluated by a nonparametric Mann–Whitney test with 95% confidence level, using GraphPad Prism 6 (version 6.01) software. The ROUT (robust regression and outlier removal) method was applied to detect outliers based on the FDR, setting the Q value to 5%. Spearman correlations for each metabolite with ACR were calculated. Receiver operating characteristics (ROC) curves analysis was carried out in the Metaboanalyst 4.0 platform [21] using the Random forest algorithm. The HumanCyc database and KEGG pathways database were used to identify metabolomic pathways.

## 3. Results

Baseline clinical characteristics from the study cohort are shown in Table 1. Except for age, no significant differences were observed between groups for gender, lipid profile, uric acid, body mass index, glycemia, eGFR or percentage of diabetics, for example.

### 3.1. Cardiorenal Metabolites Show an Altered Profile in the High-Normal Range

**Figure 1:**
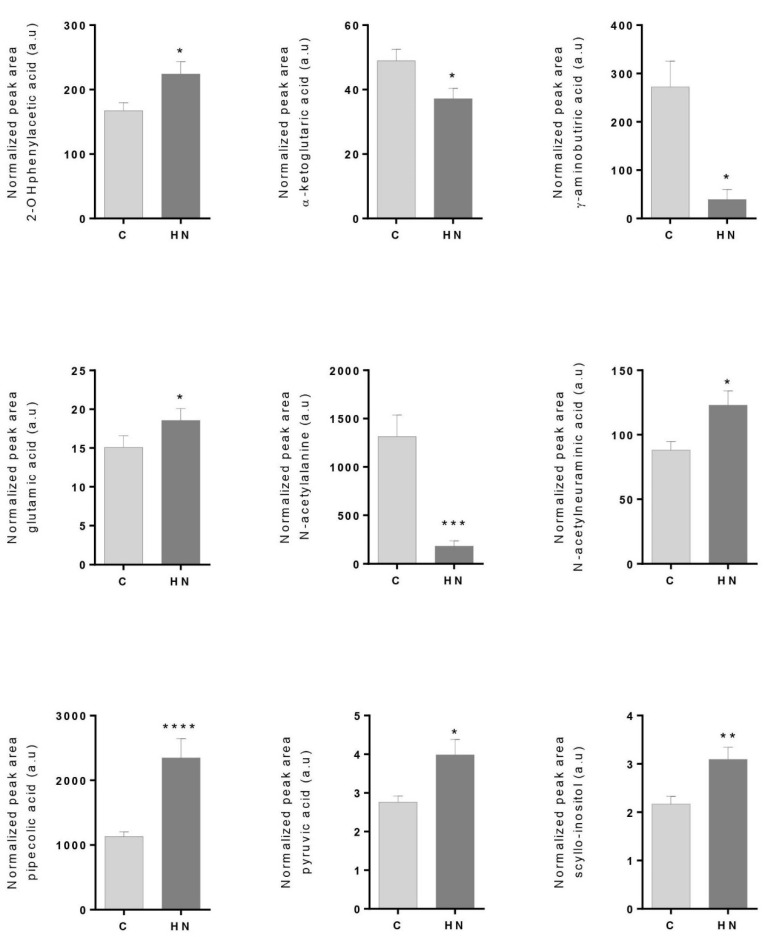
Metabolites showing significantly altered levels in urine between control subjects (C; ACR < 10 mg/g) and subjects in the high–normal range (HN; ACR = 10–30 mg/g). Error bars show standard error media. A Mann–Whitney non-parametric test (95% confidence level) was performed (* *p* value < 0.05, ** *p* value < 0.01, *** *p* value < 0.001, **** *p* value < 0.0001). Exact *p* values were 0.0142 (2-hydroxyphenylacetic acid), 0.0116 (α-ketoglutaric acid), 0.0173 (GABA), 0.0238 (glutamic acid), 0.0006 (N-acetylalanine), 0.0204 (N-acetylneuraminic acid), <0.0001 (pipecolic acid), 0.0138 (pyruvic acid) and 0.0036 (scyllo-inositol).

Among 45 measured metabolites, nine metabolites showed significantly altered abundance levels in the HN group compared to C subjects. Increased abundance levels were found for 2-hydroxyphenylacetic acid, glutamic acid, N-acetylneuraminic acid, pipecolic acid, pyruvic acid and scyllo-inositol. On the contrary, α-ketoglutaric acid, γ-aminobutyric acid and N-acetylalanine showed significantly decreased abundance levels in the HN group (Figure 1). Spearman correlation analyses showed a positive correlation with ACR for 2-hydroxyphenylacetic acid (r = 0.3802, *p* value = 0.0222), N-acetylneuraminic acid (r = 0.4569, *p* value = 0.0051), glutamic acid (r = 0.4049, *p* value = 0.0143), pyruvic acid (r = 0.3954, *p* value = 0.0170), pipecolic acid (r = 0.4047, *p* value = 0.0141) and scyllo-inositol (r = 0.4906, *p* value = 0.0024). No apparent correlation between metabolites and diabetic status was observed (Supplementary Figure S1). Significant differences were maintained when adjusted by age. In view of these data, metabolic cardiorenal outcomes were already associated with albuminuria in the normoalbuminuria condition.

**Figure 2:**
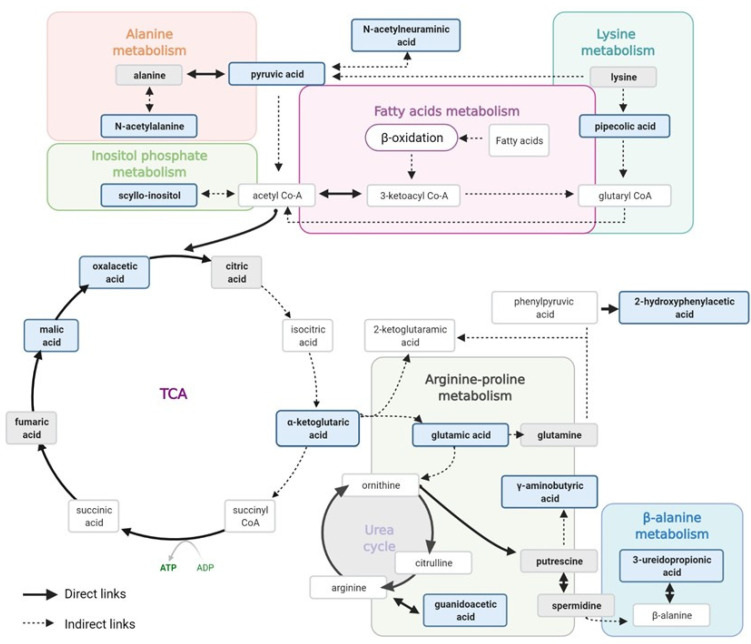
Schematic diagram of metabolic pathways showing alteration in hypertensive subjects within the high–normal range (ACR = 10–30 mg/g). Metabolites in the bold blue box represent those found significantly altered between the HN and C groups in this study and in [12]. Metabolites in the bold grey box represent those measured in this study and not altered. Other metabolites are just shown as part of the represented pathways. Created with Biorender.com.

Figure 2 shows the main metabolic pathways found here involved in early de-regulation during albuminuria development, based on the individual metabolites alterations when evaluated together. As can be seen, the metabolites of interest were related with alterations in the Argine–Proline metabolism, the TCA cycle and fatty acids (FAs) β-oxidation metabolism. The Arginine–Proline metabolism and TCA cycle were previously identified as contributors to atherosclerosis development by our group [18], as well as in our previous data on hypertensive subjects with moderately increased albuminuria [11], showing here cardiorenal damage in the normoalbuminuric condition. Energy depletion is a critical factor in CKD development and progression, and dysfunction in ATP generation by mitochondrial FAs β-oxidation in the kidney results in lipotoxicity and tubular injury [22]. In this study, these energy alterations were represented by TCA cycle and FAs β-oxidation metabolisms. FAs bound to albumin enter the proximal tubule cells taken up by membrane proteins as FABP or CD36, and are converted to acyl-CoA, which undergoes mitochondrial β-oxidation to produce acetyl-CoA for TCA cycle. Defective FAs uptake and oxidation have been linked to the development and progression of kidney diseases, and so metabolic alterations identified here point to tubular damage in an early stage of albuminuria development.

### 3.2. Renal Damage Evaluation: Altered Levels of FFAs and FABP1 in the High–Normal Range

**Figure 3:**
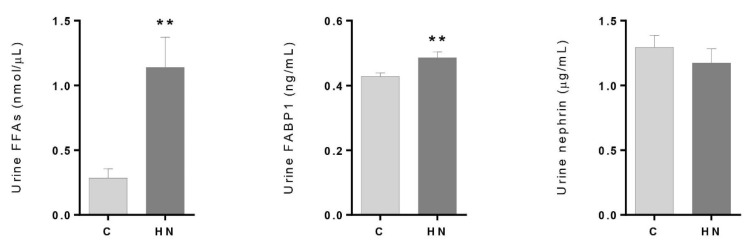
Urinary concentration levels of free fatty acids (FFAs), fatty acid binding protein 1 (FABP1) and nephrin between control subjects (C; ACR < 10 mg/g) and subjects in the high–normal range (HN; ACR = 10–30 mg/g). Error bars show standard error media. A Mann–Whitney non parametric test (95% confidence level) was performed (** *p* < 0.01).

Based on the coordinated behavior of the metabolites of interest, a potential deregulation of FAs oxidation was analyzed. When measured in urine, increased levels of FFAs were found for patients in the HN group compared to the C group (*p*-value = 0.0012) (Figure 3). Furthermore, FABP1 showed significantly increased abundance in the HN group (*p*-value = 0.0068). On the contrary, the marker of glomerular injury, nephrin, did not show a significant difference between the groups (*p*-value = 0.3995). These results together indicate early renal damage at the tubular level in association with albuminuria, even within the normal range.

## 4. Discussion

**Figure 4:**
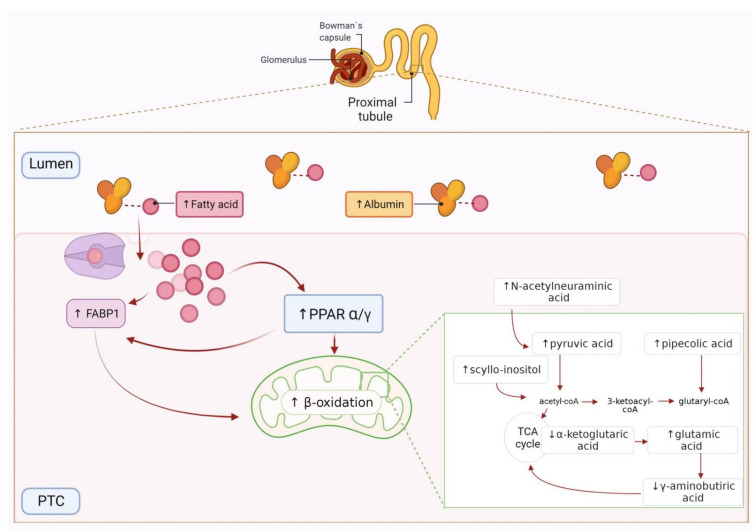
Graphic representation of fatty acids (FAs) oxidation in the proximal tubular cells (PTCs) and related metabolites. Albumin-bound FAs are taken up by FABP1 into the PTCs, transported to mitochondria and metabolized by β-oxidation. Arrows represent the metabolites abundance trend between control and HN subjects. Created with Biorender.com.

A metabolic fingerprint previously identified in association with cardiorenal risk was evaluated here in hypertensive patients within the normoalbuminuria condition. These urinary metabolites significantly differed in abundance in subjects with ACR values in the high–normal range (10–30 mg/g) compared to control individuals (ACR < 10 mg/g). This is molecular evidence of previous clinical outcomes that have linked an increased cardiorenal risk with albuminuria progression. The metabolic pathways identified here, when a coordinated response of the metabolites was studied, also suggested defective tubular reabsorption or tubular damage caused by FAs overloading in the kidney and impaired β-oxidation (Figure 4).

### 4.1. Free Fatty Acids Overload, β-oxidation and Tubular Injury in the High–Normal Range

Urinary albumin excretion is not only a marker of glomerular damage, but also of tubular injury and impaired albumin reabsorption [23]. The proximal tubule, rich in mitochondria and dependent on oxidative phosphorylation, is relevant in CKD initiation and progression [24], responding independently to albumin and its ligands [25]. Particularly, FAs bound to albumin enter the proximal tubule and are directly involved in tubular damage development [26]. The first mention of lipid nephrotoxicity and related mechanisms in progressive kidney diseases was shown in 1982 [27]. Since then, many studies have endorsed this link, reflecting mitochondrial dysfunction, impaired β-oxidation of FAs, reactive oxide species (ROS) triggers, lipid accumulation and TCA cycle alteration [28,29]. We found, here, a deregulation of TCA cycle metabolites, reduced levels of metabolites with antioxidant properties and increased levels of FFAs and FABP1 in HN subjects’ urine.

In physiological conditions, FAs are bound to albumin, filtered through the glomeruli, reabsorbed into the proximal tubules and transported to mitochondria or peroxisomes, where they are metabolized by β-oxidation. In proteinuria, increased filtration of FA-bearing albumin and/or an increased FFA/albumin ratio may contribute to proximal tubule FFA overload, favoring lipotoxicity and ROS generation and impairing proximal tubule function [25,30,31]. An increased urinary presence of FFAs, as we found here, is strongly correlated to tubule interstitial injury and proteinuria [32,33,34]. FAs up-regulate *FABP1* gene expression in the proximal tubule and accelerate its excretion into urine. When FAs are introduced in the proximal tubule cells and liberated into the cytoplasm, FABP1 lead their transport, on the one hand, to the mitochondria to be transformed into ATP by β-oxidation or, on the other hand, to the extracellular urinary space [32]. This double role of FABP1 makes it a key regulator of FAs homeostasis. Increased expression of FABP1 has been associated with increased β-oxidation of FAs, showing a protective effect against the lipotoxicity of FAs [35]. An altered mitochondrial β-oxidation was shown in early stages of normoalbuminuric diabetic kidney disease patients [36], and increased urinary levels of FABP1 suggested an increased incidence of progression of CKD and cardiovascular disease events [37,38]. The enhanced expression of FABP1 has also been related to the activation of the peroxisome proliferator activated receptor (PPAR) pathway. The PPAR regulates mitochondrial FAs oxidation, and a dose-dependent stimulation of PPAR-γ by albumin-bound FAs has been probed [39].

At the metabolic individual level, a defective reabsorption in the proximal tubule, peroxisomal stress and PPARα activity have been related to increased levels of urinary pipecolic acid in type 2 diabetes [40]. 2-Hydroxyphenylacetic acid also increased after treatment with a PPARα agonist [41]. Furthermore, a lipid-lowering effect via PPARα was described for N-acetylneuraminic acid [42]. These three metabolites had increased levels in HN subjects included here, indicating that PPAR pathway activation could potentially counteract the FAs overload [43].

On the contrary to these pieces of evidence of tubular dysfunction, the glomerular damage marker, nephrin, was not found to be altered between HN and C subjects, suggesting a higher pathological contribution from the tubule than from the glomerulus in the early stages of albuminuria development.

### 4.2. TCA Cycle Alteration and Increased ROS in the HN Range

Another consequence of mitochondrial FAs oxidation is increased ROS production and decreased pyruvate oxidation [44]. TCA cycle metabolite alterations were previously identified by our group in hypertensive patients with moderately increased albuminuria [11]. Here, and in our previous study, in normoalbuminuric subjects [12], metabolites of the TCA cycle (α-ketoglutaric acid, oxalacetic acid and malic acid) and related ones (glutamic acid and pyruvic acid) showed significant alteration in HN subjects vs. controls. α-Ketoglutaric acid is an antioxidant agent and a nitrogen scavenger involved in preventing peroxidative damage of lipids [45]. Reduced levels were observed here, meanwhile its product, glutamic acid, was increased. We have previously related a mitochondrial dysfunction in diabetic conditions [46] and in non-diabetic CKD patients, with the TCA cycle pathway being the most significantly altered [47]. α-Ketoglutaric acid was found to be markedly reduced, so too was the expression of regulating genes (*OGDH*) in kidney biopsies. These previous and current data are in line with the emerging view of CKD as a state of mitochondrial dysfunction. γ-Aminobutyric acid (GABA) also has a protective role in renal dysfunction and oxidative stress and exerts anti-inflammatory properties. It may inhibit the accumulation of lipid peroxidation products, enhances mitochondrial β-oxidation and regulates pyruvic acid oxidation [48]. Here, it was decreased in HN subjects, and previously, we found it was also decreased in young adults with CV risk factors [17].

## 5. Conclusions

In this study, we showed a urinary metabolic profile associated with high–normal albuminuria, correlating with ACR values and supporting increased β-oxidation of FAs, PPAR activation and TCA cycle dysregulation. These findings suggest tubular damage mediated by FAs overload, already taking place in the normoalbuminuria condition.

These biochemical changes, evidenced here within the normoalbuminuria range, may support hypertensive patients’ management, currently based on ACR, with new values and markers. The molecular profiles identified here could help in identifying early cardiorenal risk that requires an active therapy, identifying individual risk profiles beyond ACR and, thus, detecting normoalbuminuric subjects as potential beneficiaries of pharmacological therapy intensification or new drugs’ testing. In that situation, albuminuria could be fully normalized, indicating that the evolution of CKD has been arrested or at least delayed.

## Figures and Tables

**Table 1:** Baseline clinical data of hypertensive patients under chronic RAS suppression classified into the control group (ACR < 10 mg/g) and high–normal group (ACR = 10–30 mg/g). Data are shown as average ± standard deviation.

Characteristics	Control (C) (*n* = 21)	High–Normal (HN) (*n* = 16)	*p*-Value
Age (years)	56 ± 9	64 ± 5	0.0053
Sex (% male)	62	76	0.4912
BMI (Kg/m^2^)	31 ± 5	30 ± 5	0.5274
Cholesterol (mg/dL)	181 ± 38	166 ± 29	0.2904
Triglycerides (mg/dL)	111 ± 34	125 ± 59	0.2494
Cholesterol HDL (mg/dL)	54 ± 16	53 ± 16	0.7232
Cholesterol LDL (mg/dL)	103 ± 31	88 ± 30	0.3452
Glycemia (mg/dL)	102 ± 10	107 ± 19	0.6657
Uric acid (mg/dL)	6 ± 1	6 ± 2	0.1923
eGFR (mL/min/1.73 m^2^)	86 ± 16	85 ± 20	0.9577
ACR (mg/g)	5 ± 2	22 ± 7	<0.0001
Diabetes mellitus type 2 (%)	14	24	0.6745
SBP (mmHg)	140 ± 14	144 ± 14	0.4989
DBP (mmHg)	85 ± 8	83 ± 8	0.4707
**Antihypertensive Treatment (%)**			
iECAs	19	18	>0.9999
ARA	76	71	0.7165
Diuretic	48	53	0.7463
Calcium channel blocker	43	76	0.0453
α-blocker	19	0	0.1182
β-blocker	33	29	0.7228
**Other Treatments (%)**			
Anticoagulant	5	6	>0.9999
Lipid lowering	71	59	0.4891
Antidiabetic	10	12	>0.9999

## Data Availability

Data is contained within the article or supplementary material.

## Supplementary Materials

The following are available online at https://www.mdpi.com/article/10.3390/antiox10071100/s1, Figure S1: Spearman correlation analysis between metabolites abundance and ACR values, Table S1: Metabolites previously identified in association with cardiorenal risk and here analyzed within the normoalbuminuria condition, Table S2: Instrumental conditions for target analysis of metabolites by LC-MS/MS in selected reaction monitoring mode (SRM).
