# Supplementary figures and images for: TCA Cycle and Fatty Acids Oxidation Reflect Early Cardiorenal Damage in Normoalbuminuric Subjects with Controlled Hypertension
Source: Antioxidants (Basel). 2021 Jul 9;10(7):1100. doi: 10.3390/antiox10071100 (PMC8301016; doi:10.3390/antiox10071100)

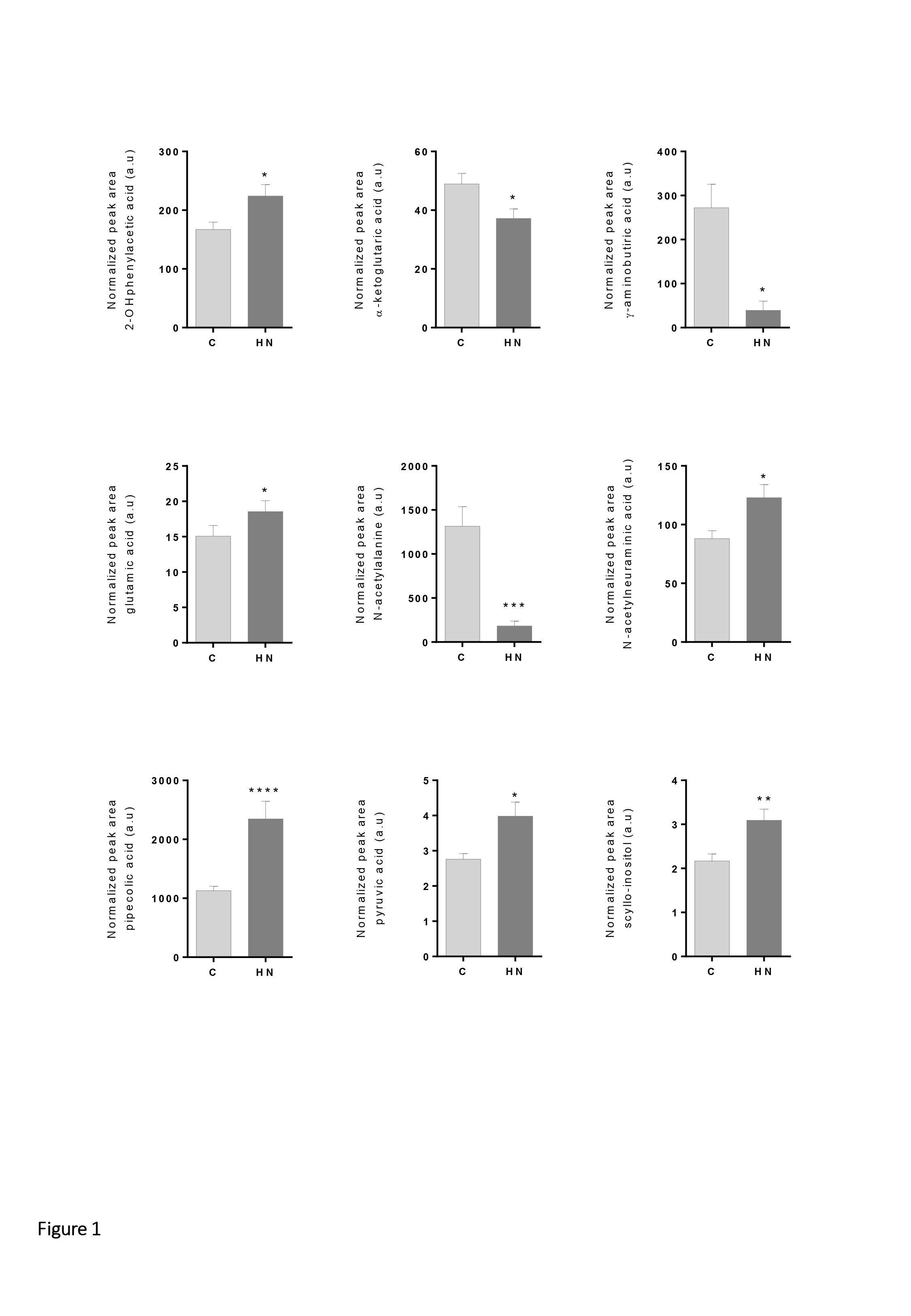

Supplement: Supplementary file 1 [file antioxidants-10-01100-s001.zip › 0001.jpg]

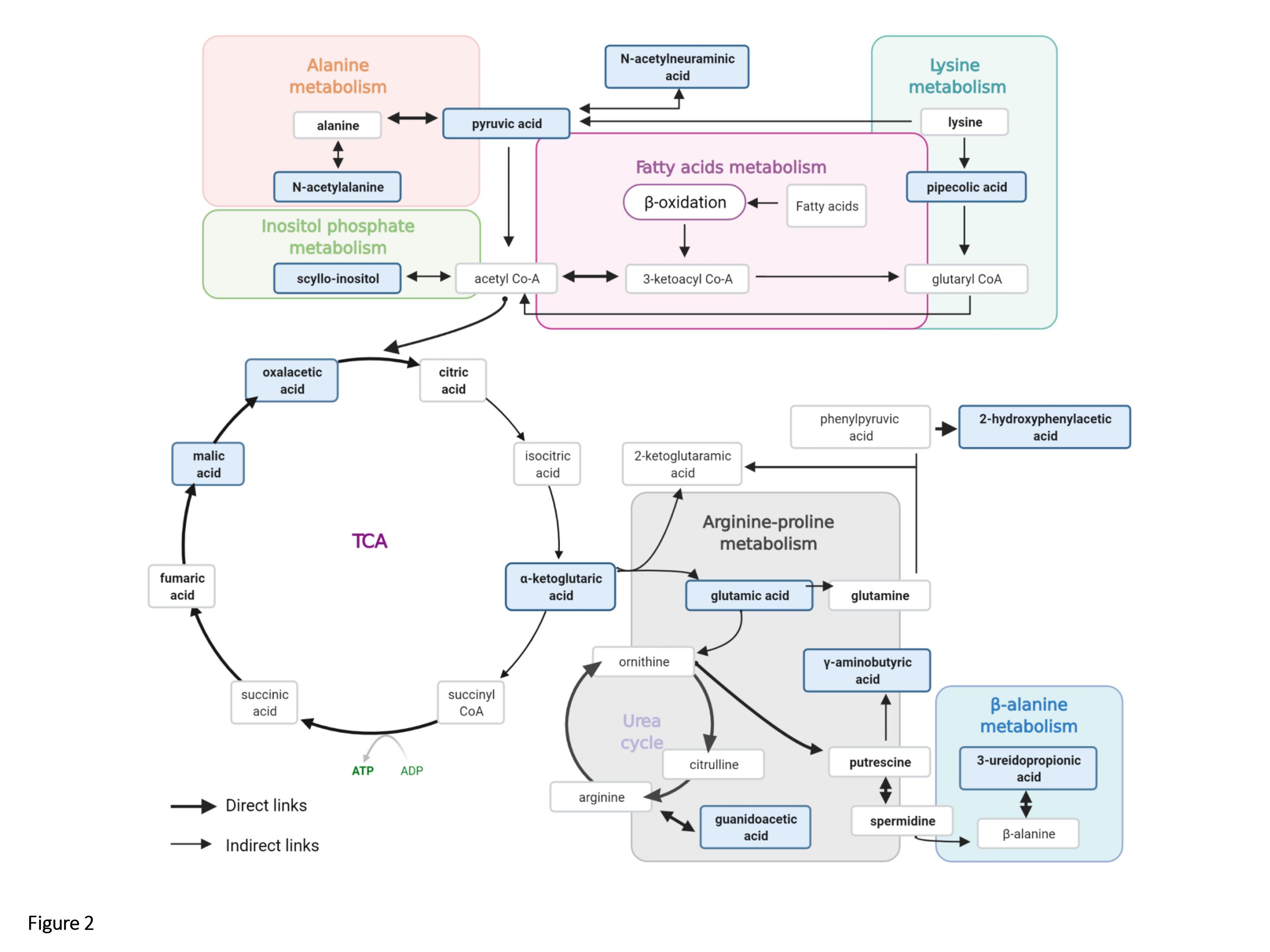

Supplement: Supplementary file 1 [file antioxidants-10-01100-s001.zip › 0002.jpg]

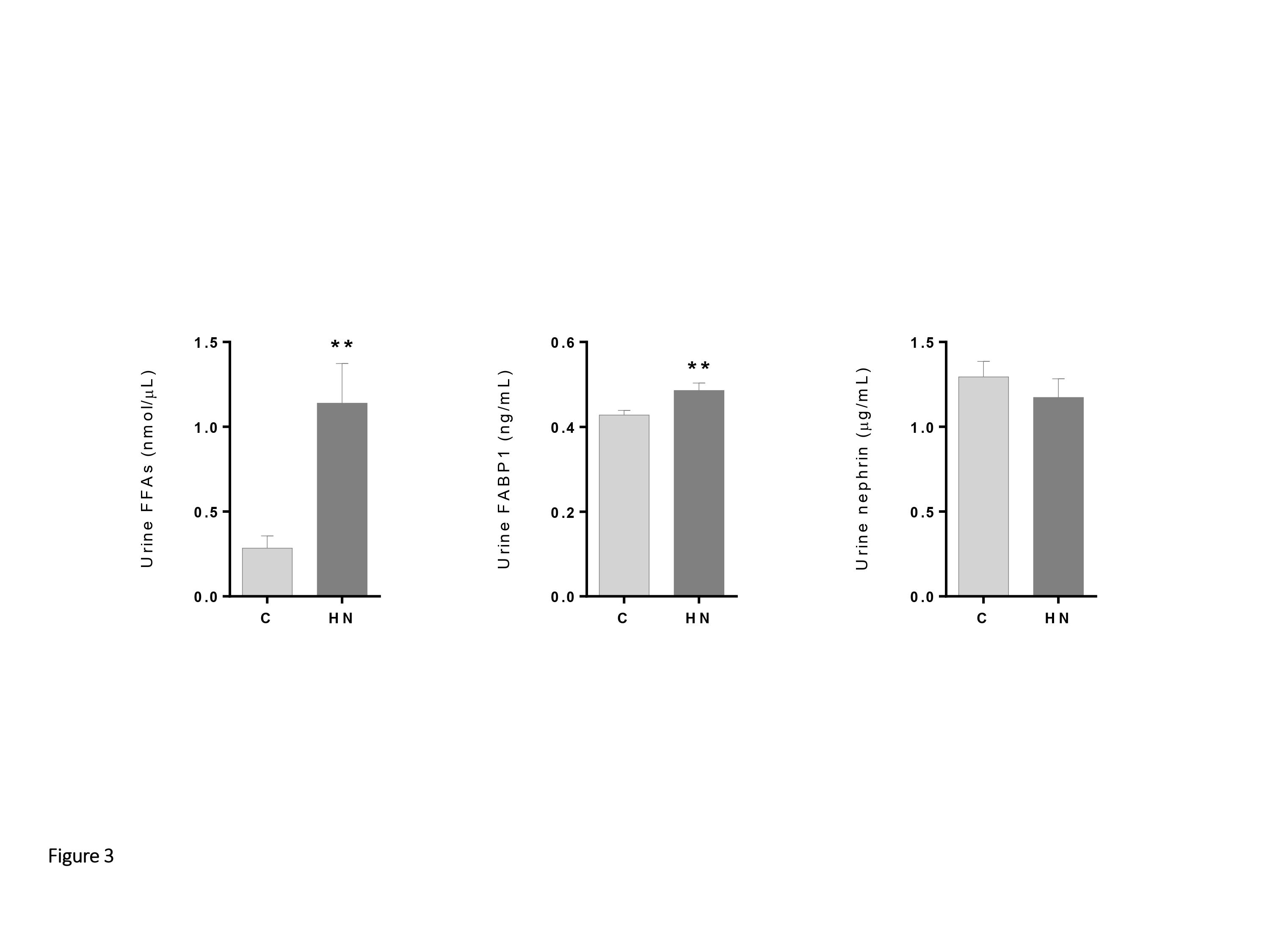

Supplement: Supplementary file 1 [file antioxidants-10-01100-s001.zip › 0003.jpg]

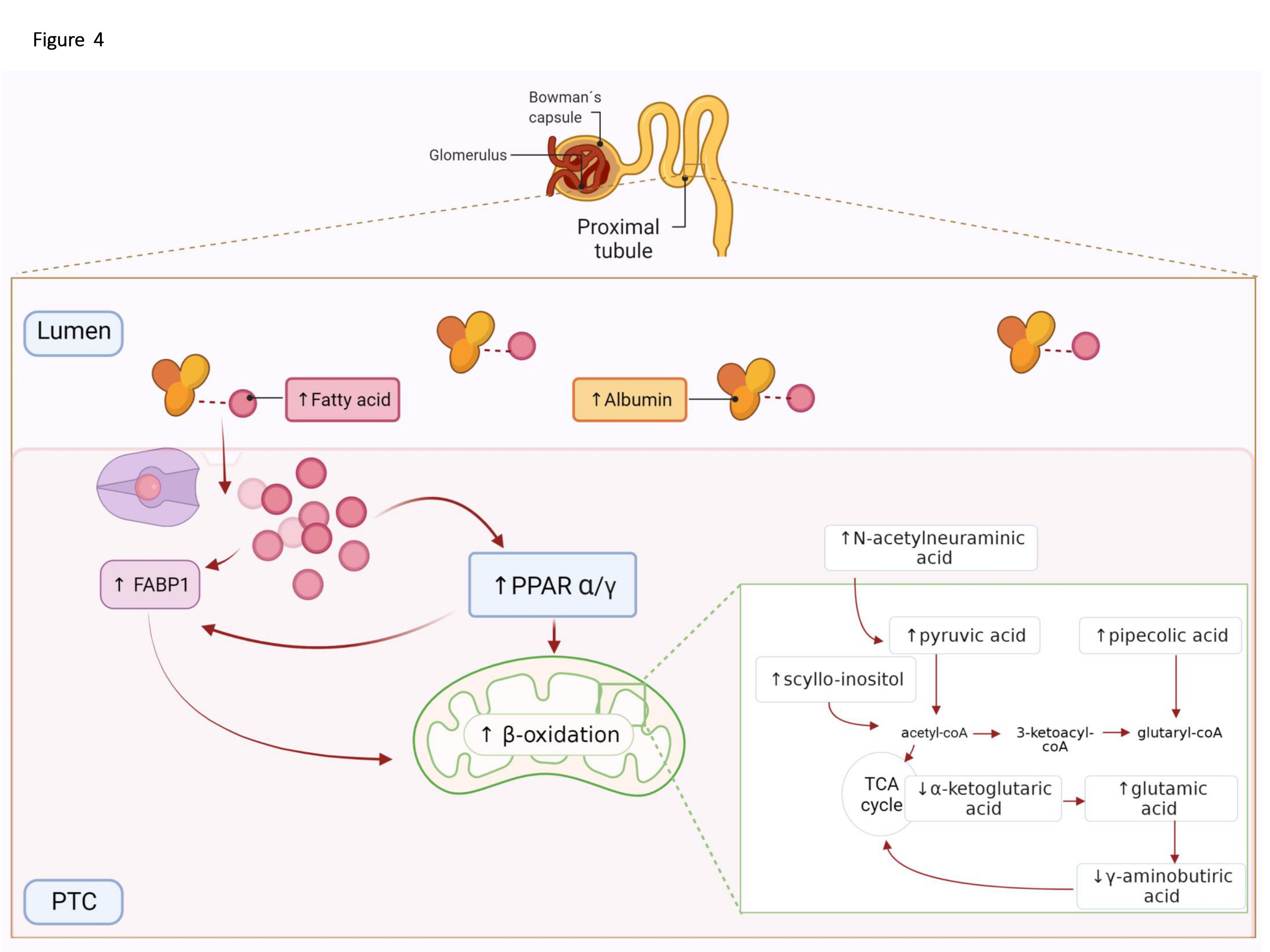

Supplement: Supplementary file 1 [file antioxidants-10-01100-s001.zip › 0004.jpg]
